# Evaluating cervical dislocation methods, without using tension on the tail, for humanely killing adult laboratory mice

**DOI:** 10.1177/00236772251332722

**Published:** 2025-06-09

**Authors:** Antony Davidge, Flaviu Bulat, Aude Vernet

**Affiliations:** Cancer Research UK, Cambridge Institute, University of Cambridge, Cambridge, UK

**Keywords:** Cervical dislocation (CD), humane killing, computerised tomography (CT), 3D imaging, animal welfare, killing

## Abstract

Cervical dislocation (CD) is a widely used method worldwide for humanely killing adult laboratory mice in accordance with national legislation, such as the Animals (Scientific Procedures) Act 1986 in the UK. However, concerns have been raised regarding the limitations and potential risks associated with CD, including a reported failure rate of up to 20% and the risk of injury to the thoracic or lumbar spine region. To address these concerns, we have adopted a CD method that avoids the use of tension on the tail or any additional tools. In this study, we detail our process of validation through self-reporting and direct observation leading up to present our implementation of computerised tomography and three-dimensional imaging software to evaluate the effectiveness and efficiency of this tail-force free CD method. Our findings reveal a 100% success rate in achieving accurate cervical dislocation without causing damage to other vertebrae, thereby providing an improved and more reliable approach to humane killing for both male and female adult laboratory mice.

## Évaluer les méthodes de dislocation cervicale, sans utiliser de tension sur la queue, pour euthanasier humainement les souris de laboratoire adultes Résumé

La dislocation cervicale (DC) est une méthode largement utilisée dans le monde entier pour tuer sans cruauté des souris de laboratoire adultes conformément à la législation nationale, telle que la loi de 1986 sur les animaux (procédures scientifiques) au Royaume-Uni. Des préoccupations ont toutefois été soulevées concernant les limites et les risques potentiels associés à la DC, notamment un taux d’échec rapporté pouvant aller jusqu’à 20% et le risque de blessure à la région thoracique ou lombaire du rachis. Pour répondre à ces préoccupations, nous avons adopté une méthode de DC qui évite l’application de tension sur la queue ou l’utilisation d’outils supplémentaires. Dans cette étude, nous détaillons notre processus de validation par auto-reporting et observation directe menant à la présentation de notre mise en œuvre de logiciels de tomographie informatisée (CT) et d’imagerie 3D pour évaluer l’efficacité et l’efficience de cette méthode de DC sans application de force au niveau de la queue. Nos résultats révèlent un taux de réussite de 100% dans la réalisation précise de la dislocation cervicale sans causer de dommages aux autres vertèbres, fournissant ainsi une approche améliorée et plus fiable de l’abattage sans cruauté pour les souris adultes mâles et femelles de laboratoire.

## Bewertung von Methoden zervikaler Dislokation ohne Zugausübung am Schwanz, zur tierschutzkonformen Tötung erwachsener Labormäuse Abstract

Die zervikale Dislokation (CD) ist eine weltweit weit verbreitete Methode zur tierschutzkonformen Tötung erwachsener Labormäuse in Übereinstimmung mit nationalen Rechtsvorschriften, wie dem Animals (Scientific Procedures) Act 1986 im Vereinigten Königreich. Es wurden jedoch Bedenken hinsichtlich der Einschränkungen und potenziellen Risiken im Zusammenhang mit der Dislokation geäußert, darunter eine gemeldete Fehlerquote von bis zu 20% sowie das Risiko von Verletzungen im Bereich der Brust oder Lendenwirbelsäule. Um diese Bedenken auszuräumen, haben wir eine CD Methode entwickelt, bei der kein Zug am Schwanz und keine zusätzlichen Werkzeuge verwendet werden. In dieser Studie beschreiben wir unseren Validierungsprozess durch Selbstbericht und direkte Beobachtung und stellen unsere Implementierung der Computertomographie (CT) und 3D Bildgebungssoftware vor, um die Wirksamkeit und Effizienz dieser ohne Zug am Schwanz ausgeführten CD Methode zu bewerten. Unsere Ergebnisse zeigen eine 100-prozentige Erfolgsquote beim Erreichen einer präzisen Dislokation der Halswirbelsäule, ohne andere Wirbel zu beschädigen, und bieten somit einen verbesserten und zuverlässigeren Ansatz für eine tierschutzgerechte Tötung von männlichen und weiblichen erwachsenen Labormäusen.

## Evaluación de métodos de dislocación cervical, sin aplicar tensión en la cola, para el sacrificio humanitario de ratones de laboratorio adultos Resumen

La dislocación cervical (DC) es un método habitualmente utilizado en todo el mundo para sacrificar de forma humanitaria ratones adultos de laboratorio de acuerdo con la legislación nacional, como la Ley de Animales (Procedimientos Científicos) de 1986 en el Reino Unido. Sin embargo, se ha expresado preocupación por las limitaciones y los riesgos potenciales asociados a la DC, incluida una tasa de fracasos comunicada de hasta el 20% y el riesgo de lesiones en la región torácica o lumbar de la columna vertebral. Para abordar estas preocupaciones, hemos adoptado un método de DC que evita el uso de tensión en la cola o de cualquier herramienta adicional. En este estudio, detallamos nuestro proceso de validación mediante autoinformes y observación directa hasta presentar nuestra implementación de tomografía computerizada (TC) y software de imágenes en 3D para evaluar la eficacia y eficiencia de este método de EC sin fuerza en la cola. Nuestros hallazgos revelan una tasa de éxito del 100% en la consecución de una dislocación cervical precisa sin causar daños a otras vértebras, proporcionando así un enfoque mejorado y más fiable para el sacrificio humanitario de ratones de laboratorio adultos, tanto machos como hembras.

## Introduction

Humane killing of animals in laboratory settings is a critical aspect of ethical research, with the intention of ensuring a painless and respectful death. Those involved in this process carry a dual responsibility of providing compassionate care throughout the animal’s life and carrying out euthanasia professionally and proficiently. However, the act of killing can lead to emotional dissonance, particularly for individuals who have a strong attachment to animals. The emotional impact is further compounded by the repetitive nature of the task, particularly when healthy animals must be killed due to experimental requirements, such as those carrying specific genotypes unsuitable for scientific studies.

Staff members often find reassurance in knowing that the methods chosen for euthanasia are deemed humane, although this comfort can be confounded when there is debate over a technique’s effectiveness.^1^ While such methods require formal training and competence assessment,^2^ with successful candidates required to be listed on the humane killing register, at least within the UK,^3^ these measures alone may not resolve concerns about their humane execution.^13,^^14^

The European Directive on the protection of animals used for scientific purposes (2010/63/EU) mandates that animals be euthanised with minimal pain, suffering and distress.^4^ However, the scientific goals of a study may dictate the method of euthanasia, leading to situations in which less humane techniques are justified to achieve research objectives. For example, physical methods may be necessary when biochemical samples are required, as an anaesthetic overdose could interfere with experimental variables. While physical methods, if executed correctly, cause minimal suffering, and do not require specialised equipment, they pose a risk of injury if performed incorrectly, making operators hesitant during their initial introduction. Cervical dislocation, one of the most widely used physical methods, raises welfare concerns due to the reported high failure rates^5^ and limited evidence suggesting they may not immediately terminate brain activity or consciousness. Recent publications support the need for new refinements and explore the potential use of tools to improve accuracy and ensure cervical dislocation in laboratory rodents.^6^ We explored previously unreported methods aimed at minimising tension on the tail to assess their potential as a humane alternative for euthanasia. Specifically, we investigated whether these methods could serve as viable substitutes for the more traditional approach of applying tension to the tail and force to the cervical region. This study was motivated by the need to identify more humane and effective techniques, particularly considering Carbone’s research,^5^ we utilised advanced imaging technology to confirm successful cervical dislocation. Computerised tomography (CT) imaging was selected for its clinical application in producing detailed images of the body. Additionally, the use of three-dimensional (3D) imaging software was prioritised for its accessibility to lay reviewers, as opposed to the traditional cross-sectional X-ray slices of bones, blood vessels and soft tissues, which demand a higher level of knowledge, experience and expertise to interpret effectively.

The traditional method for cervical dislocation (CD) in laboratory settings involves the use of tools, such as a pen, pencil, or similar object, placed behind the ears at the base of the skull to apply slight forward and downward pressure while pulling the tail to dislocate the cervical vertebrae.^7^ Alternatively, tension can be directly applied to the tail without using tools while keeping the head stationary.

## Methods

We present a modified CD technique that aims to improve on traditional methods by eliminating the use of tools and force on the tail, with the goal of standardising a more effective and humane approach for CD in laboratory animals. The focus of this study is to improve the reported failure rate of approximately 20%, working towards achieving 100% accuracy in cervical dislocation. At the Cambridge Institute, the approved method for cervical dislocation prohibits the use of tools or tension on the animal’s tail. During the procedure, animals are restrained either by the scruff or by the tail, and depending on the handler’s preference the cervical dislocation itself can be performed using one of three techniques – push, pinch, or roll. While these methods share significant similarities in that they all involve the application of direct pressure to the cervical region, they differ subtly in execution. The push technique emphasises a forward motion, the pinch focuses on targeted compression, and the roll incorporates a rotational movement. Importantly, in all methods, the tail remains tension-free, ensuring consistency in humane practice. In the first technique, a ‘pinch’ is performed by placing the fingers either side of the cervical area and bringing them together to exert force. The second method involves a ‘push’, in which the fingers are positioned on either side of the head and used to push the head forward. The third technique is a ‘roll’, in which the thumb is employed to apply forward pressure to the head through a slight rolling motion. The tests we apply for successful CD are a clear separation of a 3D image for non-CT experts and clinically approved measurements of gaps for the CT expert. To evaluate the efficacy of the modified CD technique, we conducted a three-stage investigation. The methods are detailed under the following subheadings: research design, ethical approval, setting, participants, procedures and interventions, animals, instruments and measures, data collection and data analysis.

### Research design

This study involved a three-stage investigation aimed at evaluating the efficacy of a CD technique. The investigation stages included a period of self-reporting, direct observation of an individual and the final stage imaging of animals following CD by a number of different operators over a period of time (see Tables 1 and 2).

**Table 1.** Cohort details.

	Cohort 1	Cohort 2	Cohort 3	Cohort 4	Cohort 5	Cohort 6
No. of mice	12	9	12	12	12	11
Sex (M/F)	4/8	1/8	6/6	8/4	6/6	11/0
Age (days) mean	175	214	87	75	75	396
Age (days) range	112–267	104–529	82–94	37–110	49–173	376–445
No. of operators	4	3	2	2	2	2
Operator ID	1,2,3,4	5,6,7	8,9	10,11	12,13	9,13
Euthanasia method	CD	CD	CD	CD	CD	CD/control

**Table 2.** Operator details.

Operator ID	Experience (years)	Sex	Occasions	No. of animals	Prefer
1	5	F	One (cohort 1)	3	Push
2	1	M	One (cohort 1)	3	NR
3	5	F	One (cohort 1)	3	Push
4	3	F	One (cohort 1)	3	NR
5	10	F	One (cohort 2)	3	Pinch
6	10	M	One (cohort 2)	3	Roll
7	15	M	One (cohort 2)	3	Pinch
8	15	F	One (cohort 3)	6	Push
9	10	F	Two (group 3, 6)	11	Push
10	10	M	One (cohort 4)	6	Roll
11	3	M	One (cohort 4)	6	NR
12	10	M	One (cohort 5)	6	Pinch
13	1	F	Two (cohort 5, 6)	10	NR
Controls			One (cohort 6)	2	NA

NR: Not recorded at time of event.

### Ethical approval

The study was approved by the Cambridge Institute’s Animal Welfare and Ethical Review Body. All activities involving animals were conducted in accordance with the Animals (Scientific Procedures) Act 1986, Amendment Regulations 2012, and the EU Directive 2010/63/EU. Welfare considerations and the principles of the 3Rs (replacement, reduction, refinement) were adhered to throughout the study to minimise any potential suffering to animals.

### Setting

The study was conducted at the CRUK Cambridge Institute, utilising BRU (Biological Resource Unit) staff members and animals scheduled for euthanasia following colony management.

### Participants

Thirteen different operators with experience ranging from 1 to 15+ years participated in the study. The operators were BRU staff members available at the time of euthanasia, all had been assessed as competent and reverified for the no tail tension CD technique. Only those operators that had 10+ years (*n* = 7) would have had experience of tools or tail tension technique, with *n* = 6 having no experience of tension on the tail or tools.

### Procedures and interventions

Stages 1 and 2 served as complementary evaluation tools, designed to provide a layered approach to assessment before advancing to stage 3. These initial stages allowed for preliminary evaluation of key indicators and criteria, ensuring that only cases meeting predefined thresholds progressed to the larger, more involved stage 3. In particular, the observations and outcomes from stages 1 and 2 were critical in determining the readiness and suitability for further evaluation, effectively functioning as a go/no go decision point within the overall process.

#### Stage 1

Self-reporting by staff performing CD during a 30-day period in June 2021. Staff reported if they observed any animal gasping after carrying out the procedure, considering gasping as an indicator of a failed attempt. Approximately one thousand animals were estimated to be involved based on usage records for the year.

#### Stage 2

An experienced technician was directly observed while performing 60 CD operations in approximately 30 minutes. An independent observer monitored success rates based on the presence or absence of gasping; observations were carried out until the onset of rigor mortis.

#### Stage 3

Imaging a total of 68 animals, 66 animals that underwent CD and two controls killed by a rising levels of carbon dioxide, to provide baseline CT images of the intact spine. The killing and imaging took place on six separate occasions with each day being identified as a cohort, involving a total of 13 different operators. Observation was continuously carried out until the onset of rigor mortis (see Table 1 and Table 2). The collection of CT images was conducted by a specialist with extensive experience in CT imaging, ensuring accuracy and consistency. Additionally, the individuals involved in the collection of samples and performing the procedures were independent, minimising any potential bias and maintaining the integrity of the study.

### Animals

The animals were housed in Tecniplast GM500 ‘green line’ cages with aspen bedding, a cardboard tunnel, pulped virgin cotton fibre nesting material, and an aspen chew block. They were provided with a commercially available diet (LabDiet PicoLab Rodent 20 – 5053 or 5R58) and were free from mouse pathogens as determined by routine health screening carried out according to FELASA guidelines^9^.

Animals were acquired from various research groups, comprising different strains and sexes, most of which were on a C57BL/6J background. The animals used in stages 2 and 3 were animals randomly allocated using a colony management database, without additional selection criteria such as weight, age or sex. Allocation followed a first come, first served approach based on availability within the colony, and while no deliberate effort was made to balance distribution, the sample size was expected to provide sufficient representation across variables. Animal were in good health, had not previously been used for experimentation, and were scheduled for culling due to colony management or being deemed unsuitable for other research projects. Animals involved in stage 1 predominantly would have been killed due to colony management, there would also have been a number killed at end of experiment or ill health, estimated at 8%.

### Instruments and measures

Mediso nanoScan CT system: Used for imaging animals from stage 3 to assess damage to the bone structure of the spine and neck.

‘3DSlicer’ software: Used for visualisation of CT scans and creating 3D models.

Self-reporting forms and observational checklists: Used in stages 1 and 2 for monitoring success rates and the presence or absence of gasping.

### Data collection

All data from stages 1, 2 and 3 relied on observing and recording the presence or absence of gasping during the procedure. This was achieved through a combination of self-reporting and direct observation, documented on paper records, with stage 3 further incorporating imaging to enhance data collection and analysis. All cadavers from stage 3 were imaged using a Mediso nanoScan CT system, with image projections reconstructed at 118 μm voxel resolution. The images were processed and viewed using ‘3DSlicer’ software to create 3D models, allowing for detailed examination of the spinal structures^8^.

### Data analysis

By systematically recording the presence or absence of gasping through self-reporting and direct observation, the data provided a simple means of assessing the effectiveness and reliability of the procedure at each stage. Reviewers were blinded to animal and operator details and scored images independently. Initial review of the 3D images was conducted by two facility personnel with no previous experience in examining CT images using ‘3DSlicer’ software: used for visualisation of CT scans and creating 3D models. Their findings showed remarkable consensus in identifying suitable breaks, with only one animal in 68 being in disagreement. A formal statistical sample size calculation was not carried out for this study. However, we consulted with statisticians to determine the approximate number of animals required to observe the type of error reported in Carbone’s^5^ study. This study was designed as a proof-of-concept and descriptive investigation, aiming to explore the feasibility of the approach. Establishing statistical significance or robust conclusions about efficacy would require a larger, multi-facility study to achieve comprehensive and definitive results.

To validate the findings further, an additional reviewer – an expert with CT scan imagery assessed the CT images. The CT scans in DICOM format were processed using Vivoquant 4.0 (Patch 1, inviCRO, 2017) and saved as images in .GIF and .PNG format.

We have used a common technique to determine cervical dislocation status by measuring the ratio of basion to C1 and opisthion to C1. The exact measurements were taken from the tip of the basion to the spinolaminar line of C1 and dividing it by the distance from the tip of the opisthion to the midpoint of the posterior aspect of the anterior arch of C1. We have used the same threshold to this ratio (<0.9) used in clinic to determine normal versus dislocated.^10^ The ratio threshold quoted has been widely accepted and adopted in clinical practice.^11^ The data generated this way are quantitative and reliable.

## Results

The first stage of our investigation utilised Carbone’s findings, which suggest that if an animal gasps following an attempted CD, the procedure should be considered unsuccessful. This phase served as a preliminary screening (‘go/no go’) before proceeding to the more labour-intensive imaging session. Staff were asked to self-report any observed gasping incidents while performing CD over the course of one month. Although the exact number of animals euthanised or the number of operators involved during this period was not recorded, our facility typically processes between 1200 and 1500 animals monthly, with over 90% euthanised by CD. During the observed month, no gasping incidents were self-reported, leading us to conclude that the CDs were carried out correctly without complications.

Stage 2 involved the direct observation by the lead author of a single experienced operator performing CD on 60 animals. Similar to stage 1, no imaging was planned, and the procedure’s success was judged solely on the presence or absence of gasping following the procedure and until rigor mortis set in. No gasps were observed.

**Figure 1.**
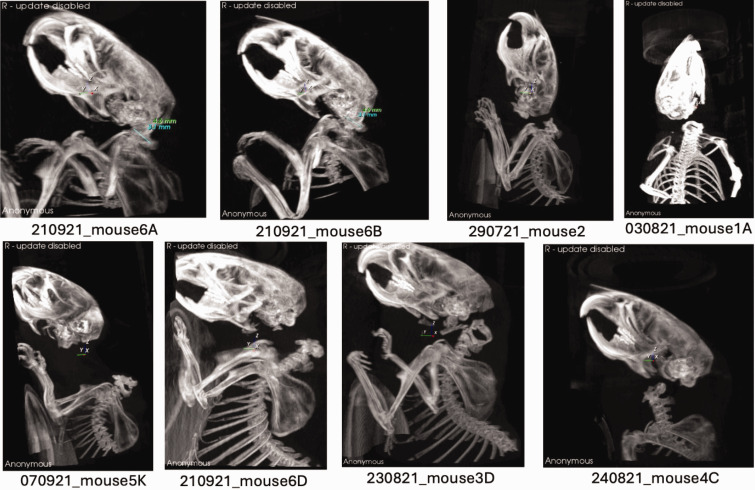
A selection of representative maximum intensity projection (MIP) computerised tomography (CT) images from each cohort are shown. Control mice 6A and 6B exhibit a Powers ratio of less than 0.9, indicating normal atlanto-occipital alignment. Mice 2, 1A, 5K, 6D, 3D and 4C, representative of their respective cohorts, clearly demonstrate cervical dislocation.

For stage 3, both the observation of the procedure (monitoring for gasping) and CT imaging were conducted. As in the previous stages, no gasping was observed in all animals in cohorts 1–6 (Table 2). Successful CD was confirmed through the analysis of CT scan images (Figure 1), using data from stage 3 cohorts.

Operator age, experience and sex did not appear to affect the results, as experience ranged from a few months to several years (Table 2). However, it is important to note that all operators had undergone initial assessment and were subject to routine re-verification.

## Discussion

The study design evolved through multiple stages to address comprehensively concerns regarding the accurate reporting of events and to evaluate the efficiency of a specific method for CD. In stage 1, self-reporting was utilised to gather data on the absence of gasping following CD procedures. This approach relied on operatives self-reporting, and therefore it is acknowledged that there exists the potential risk of underreporting due to their knowledge of the study’s objective. To mitigate this underreporting concern, stage 2 was implemented, introducing direct observation of an experienced operative performing 60 CD procedures within a defined time period. This step aimed to provide a more objective assessment of the possible occurrence of gasping and/or underreporting as according to Carbone we could expect to up to 12 animals to gasp and served as another go/no go criterion for further investigation. Both stages 1 and 2 contributed to a comprehensive evaluation of the lack of the gasping phenomenon and its potential implication as an indicator of unsuccessful completion of CD.

The primary focus of the study, stage 3, involved employing CT scans to assess the efficacy of a non-tension on the tail method for CD. The study aimed to determine whether this method effectively reduced the reported 20% failure rate documented by Carbone et al.^5^ Although this study was focused on the technique and not individual competency, it must be acknowledged that there is some debate regarding the ‘Hawthorne effect’, in which operators believe that when their skills are being assessed concentrate on their technique simply because it is being evaluated or studied.^12^ Debriefings with operators showed a genuine interest in being reassured that the technique they chose brought about a quick and humane death with no suffering.

Utilising advanced imaging and modelling software to create 3D images allowed for a more comprehensive and user-friendly analysis of the scanned area for the non-CT experienced researchers. This approach proved effective in assessing bone structures and identifying spinal fractures. The use of 3D technology greatly contributed to the reliability of the study’s results.as confirmed by the specialist.

In certain images, we observed ‘damage’ to the base of the skull. However, for the purpose of this investigation, we considered a procedure successful if it resulted in no gasp and spinal cord severance within the cervical area. It is not possible to determine whether the fracture occurred post mortem, as this may be attributed to an operator’s preference for creating a noticeable gap when executing the technique. There appeared to be no discernible correlation between the choice of technique or the operator in occurrence.

In summary, the study design demonstrated a progression through various stages to address the challenges of accurate reporting to assess the efficiency of a novel CD method. The incorporation of CT imaging and comparison with prior research findings added depth to the investigation. Despite potential limitations in sample size, the study’s approach provides valuable insights into the complex realm of cervical dislocation techniques and their practical implications. Further research with larger and more diverse samples could potentially validate and expand on the observed results.

The study presented a modified CD technique as an alternative to traditional methods, which may not align with some facilities’ current practices. It is acknowledged that operators in these facilities may find it uncomfortable to investigate or question their existing techniques, especially when they believe they are humane. However, it is crucial for all operators to consider carefully the various factors involved in CD to ensure the highest level of animal welfare and compliance with legal and moral requirements.

The published literature on CD highlights legitimate concerns about the success of the technique, indicating that more resources should be allocated to explore and develop more humane approaches. Fortunately, making small changes to the physical technique may not significantly impact resources, but the potential benefits to animal welfare are substantial. Effective communication is essential to ensure the successful implementation of the modified technique. By highlighting the advantages and minimal changes required, all stakeholders can be informed of the benefits for animal welfare.

It is strongly encouraged that facilities choosing to adopt the modified technique publish their transitioning and implementation processes, including the challenges and successes encountered. This will enable other facilities to make informed decisions and ensure continuous improvement in animal welfare practices.

Overall, the study’s findings advocate for continuous improvement in CD techniques and the adoption of more humane and effective methods in laboratory animal euthanasia. By promoting open communication, sharing experiences and learning from one another, the scientific community can collectively enhance animal welfare standards and further contribute to the 3Rs principles (replacement, reduction and refinement) in animal research. In conclusion, current scientific publications demonstrate an unacceptable failure rate for traditional CD methods, prompting facilities to be prepared to address these concerns with evidence-based practices rather than relying solely on belief. The study provides reassurance that a humane and effective alternative exists, and it is hoped that the findings will guide and inform decision-making processes in developing and implementing best practices for animal welfare. However, it is acknowledged that the study represents only a single facility, and a larger multicentre prospective study investigating various techniques could provide even greater confidence in the results.
